# Efficacy of immune checkpoint inhibitors in advanced non‐small cell lung cancer patients with *KRAS* mutations: A network meta‐analysis

**DOI:** 10.1111/crj.13745

**Published:** 2024-04-02

**Authors:** Lin Zhang, Wei Chen, Hongtao Wei, Junxian Yu

**Affiliations:** ^1^ Department of Pharmacy, Beijing Friendship Hospital Capital Medical University Beijing China; ^2^ Department of Pharmacy Emergency General Hospital Beijing China

**Keywords:** immune checkpoint inhibitors, KRAS mutation, network meta‐analysis, non‐small cell lung cancer, programmed cell death ligand‐1, programmed cell death‐1

## Abstract

**Objective:**

Previous studies have shown that immune checkpoint inhibitors can improve the survival of patients with advanced non‐small cell lung cancer with *KRAS* mutations; however, there is a lack of comparisons between treatment regimens associated with immune checkpoint inhibitors, and our study aims to compare several treatment parties to find a more effective treatment regimen.

**Method:**

A comprehensive literature search was conducted across multiple databases, namely PubMed, Web of Science, Embase, and Cochrane Library, to identify relevant studies. The screened studies were thoroughly examined, and data were collected to establish a Bayesian framework. The study focused on two primary endpoints: overall survival (OS) and progression‐free survival (PFS). Data analysis and graphical plotting using R software and Revman (version 5.3). It is worth mentioning that the study protocol was registered with the International Prospective Registry for Systematic Reviews, ensuring transparency and adherence to predetermined protocols (CRD42022379595).

**Result:**

In total, our analysis included six RCTs involving 469 patients with *KRAS* mutations. Among these patients, 224 received chemotherapy, while 245 were treated with immune checkpoint inhibitors. Meta‐analysis results showed that the addition of ICIs could significantly improve OS and PFS (0.69, 95% CI 0.55, 0.86; 0.57, 95% CI 0.42, 0.77). The results of the network meta‐analysis showed that Pembrolizumab could improve OS (HR 0.42, 95% CI 0.22–0.80) and Pembrolizumab emerged as the most effective treatment option for enhancing OS in patients (SUCRA 65.03%). Additionally, pembrolizumab in combination with chemotherapy showed improvement in PFS (HR 0.47, 95% CI 0.29–0.76).

**Conclusion:**

Our analysis found that among advanced NSCLC patients with *KRAS* gene mutations, first‐line treatment with pembrolizumab alone demonstrated greater efficacy. Similarly, second‐line treatment with nivolumab alone was found to be more effective in this patient population. However, the sample size of this study was limited, Therefore, additional clinical data is necessary to validate this finding in subsequent research.

## Graphical abstract

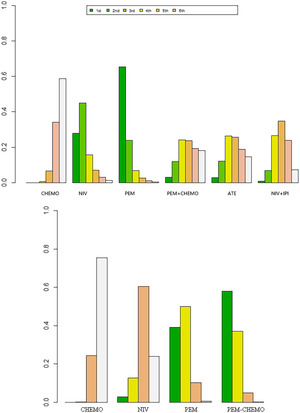

## 1 INTRODUCTION

Lung cancer, being one of the most devastating and lethal malignancies globally, particularly non‐small cell lung cancer (NSCLC), represents the predominant histological subtype among lung cancer cases.^1^ Previous research has consistently demonstrated that the *KRAS* gene plays an important role as a causal gene in NSCLC and is frequently mutated. Mutations in the *KRAS* gene are particularly common in people who are current or former smokers.^2^, ^3^ At present, platinum‐doublet chemotherapy remains the standard first‐line treatment of choice for NSCLC patients who do not have driver gene mutations. Targeted therapies are mainly focused on patients with *EGFR* mutations, *ALK* mutations, and *ROS1* mutations. Additionally, significant progress has also been made in targeted therapies for other less common driver gene mutations. Although *KRAS* gene mutations are common, they activate different signaling pathways depending on the specific mutation site, which has led to slow progress in targeted therapy research for NSCLC patients with *KRAS* gene mutations.^4^, ^5^, ^6^ Patients with NSCLC who have *KRAS* mutations generally exhibit a lower overall survival rate compared to those with *KRAS* wild‐type.^4^ Therefore, there is a necessity to explore treatment options that can offer improved clinical benefits to NSCLC patients with *KRAS* mutations.^7^, ^8^, ^9^ Tumor development is intricately interconnected with the immune system. The immune checkpoint molecules, including cytotoxic T‐lymphocyte antigen‐4 (CTLA‐4) and programmed death receptor‐1 and its ligand (PD‐1/PD‐L1), play a significant role in facilitating immune evasion by tumor cells. Immune checkpoint inhibitors (ICIs), which specifically target CTLA‐4 and PD‐1/PD‐L1, have demonstrated the potential to restore immune cell activity against tumor cells and mitigate immune evasion. These inhibitors enhance the immune response and inhibit tumor growth by modulating interactions between tumor cells, antigen‐presenting cells, and T cells.^10^, ^11^ Recently, ICIs have shown promising clinical efficacy and significantly improved patient outcomes in NSCLC. Previous research has also suggested that patients with *KRAS* mutations are more likely to benefit from immunotherapy than those with wild‐type *KRAS*.^12^, ^13^, ^14^, ^15^ Most studies have focused on examining *EGFR* mutations, with little attention paid to *KRAS* mutations. However, recent evidence suggests that NSCLC patients with *KRAS* mutations may experience greater benefits from ICIs than those with *EGFR* mutations.^16^ Clinical studies have collected survival data on patients with *KRAS* mutations in the context of ICIs. However, there is a lack of comprehensive pooled analyses and systematic comparisons across various classes of ICIs. To address this, we conducted a network meta‐analysis (NMA) to evaluate the efficacy and ranking of different treatment regimens involving ICIs in NSCLC patients with *KRAS* mutations. The aim was to provide useful insights for developing clinical treatment strategies that are tailored to NSCLC patients with *KRAS* mutations identified through genetic testing.

## 2 MATERIALS AND METHODS

### 2.1 Literature search strategies

Two researchers conducted separate searches on Web of Science, the Cochrane Library, Embase, and PubMed, until October 8, 2023. In case of any discrepancies, a third researcher resolved the conflicts. The study adhered to the guidelines outlined in the Preferred Reporting Items for Systematic Reviews and Meta‐Analyses (PRISMA).^17^ Additionally, the research protocol was registered with the International Prospective Registry for Systematic Reviews, with the registration code: CRD42022379595. The search terms were extracted based on the PICOS principles, including three subject terms (“immune checkpoint inhibitors,” “non‐small cell lung cancer,” “randomized controlled trial”) and eleven specific names of immune checkpoint inhibitors (“Nivolumab,” “Pembrolizumab,” “Avelumab,” “Atezolizumab,” “Tremelimumab,” “Camrelizumab,” “Durvalumab,” “Sintilimab,” “Ipilimumab,” and “Sugemalimab”).

### 2.2 Inclusion criteria

1. Patients with histologically and cytologically confirmed metastatic or advanced NSCLC.
2. Hazard ratios (HR) with 95% confidence intervals (CI) were reported for OS and progression‐free survival (PFS) in those patients who had *KRAS* mutations.
3. Phase II or III RCTs.
4. ICIs were employed in the treatment.

### 2.3 Exclusion criteria

1. Reviews, case reports, letters, meta‐analyses, comments, or summaries.
2. RCTs containing the same patient group.
3. Postoperative adjuvant therapy, neoadjuvant therapy, or a combination of radiotherapy.

### 2.4 Data extraction and data analysis

We extracted various information, including the first author, study name, number of patients, interventions, year of publication, and risk ratios with 95% CI for OS and PFS. Each included study was assessed for risk of bias using the Cochrane Risk of Bias Assessment Tool, categorized as low risk, unclear, or high risk.

Statistical analyses were performed using the “JAGS” and “GeMTC” packages in R, as well as RevMan (version 5.3). The study endpoints were PFS and OS. A fixed‐effects model with 2000 simultaneous iterations on three independent Markov chains was employed. Each chain underwent 50 000 sample iterations. The treatments were ranked from best to worst using the probability ranking command, and the two‐sided difference was assessed for statistical significance (α < 0.05). To evaluate study heterogeneity, we utilized I^2^ values. I^2^ values below 25% indicate low heterogeneity, suggesting the use of a fixed‐effects model.

## 3 RESULT

### 3.1 Literature search results

**FIGURE 1:**
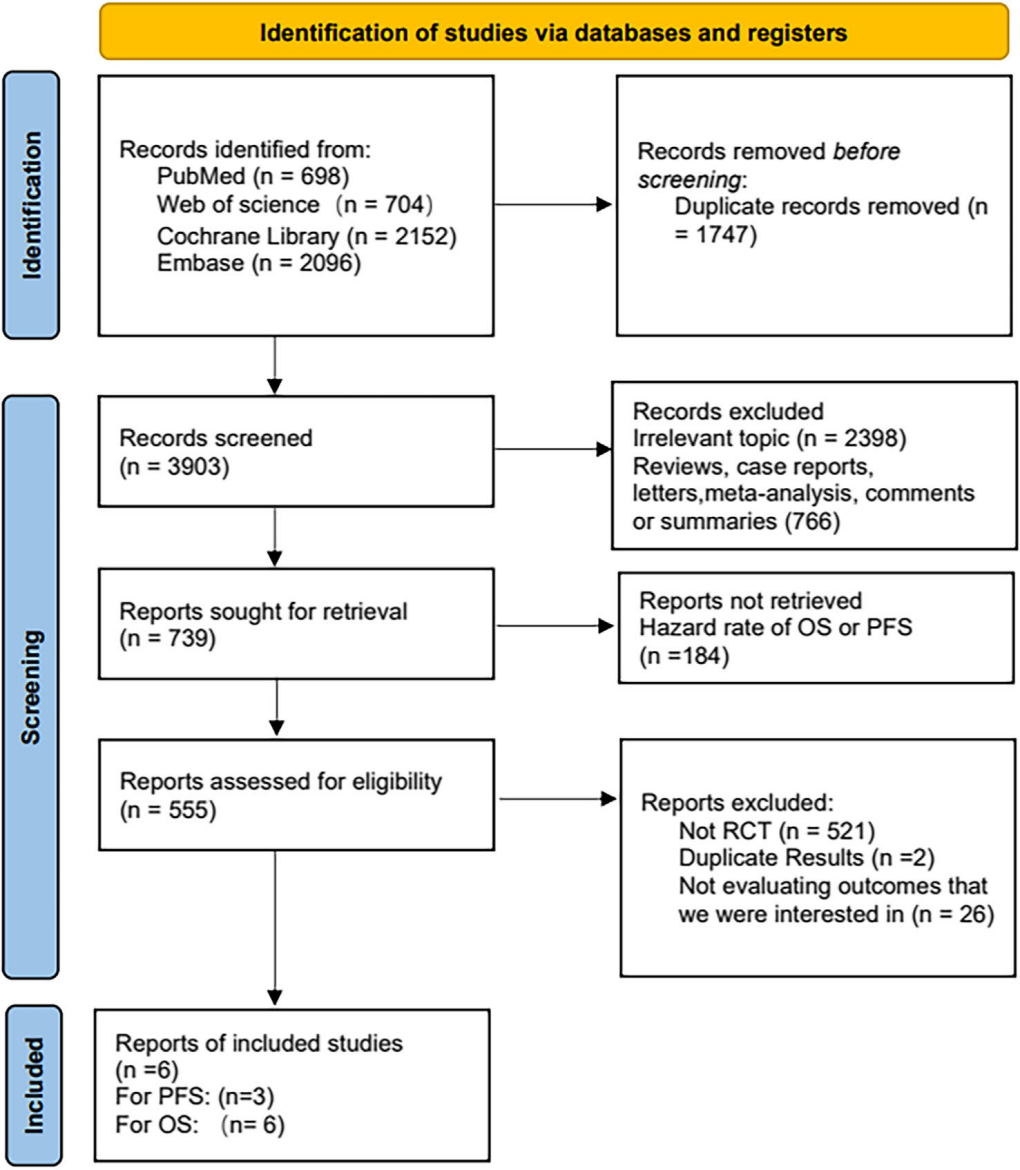
Describes a flowchart for retrieving and screening.

**FIGURE 2:**
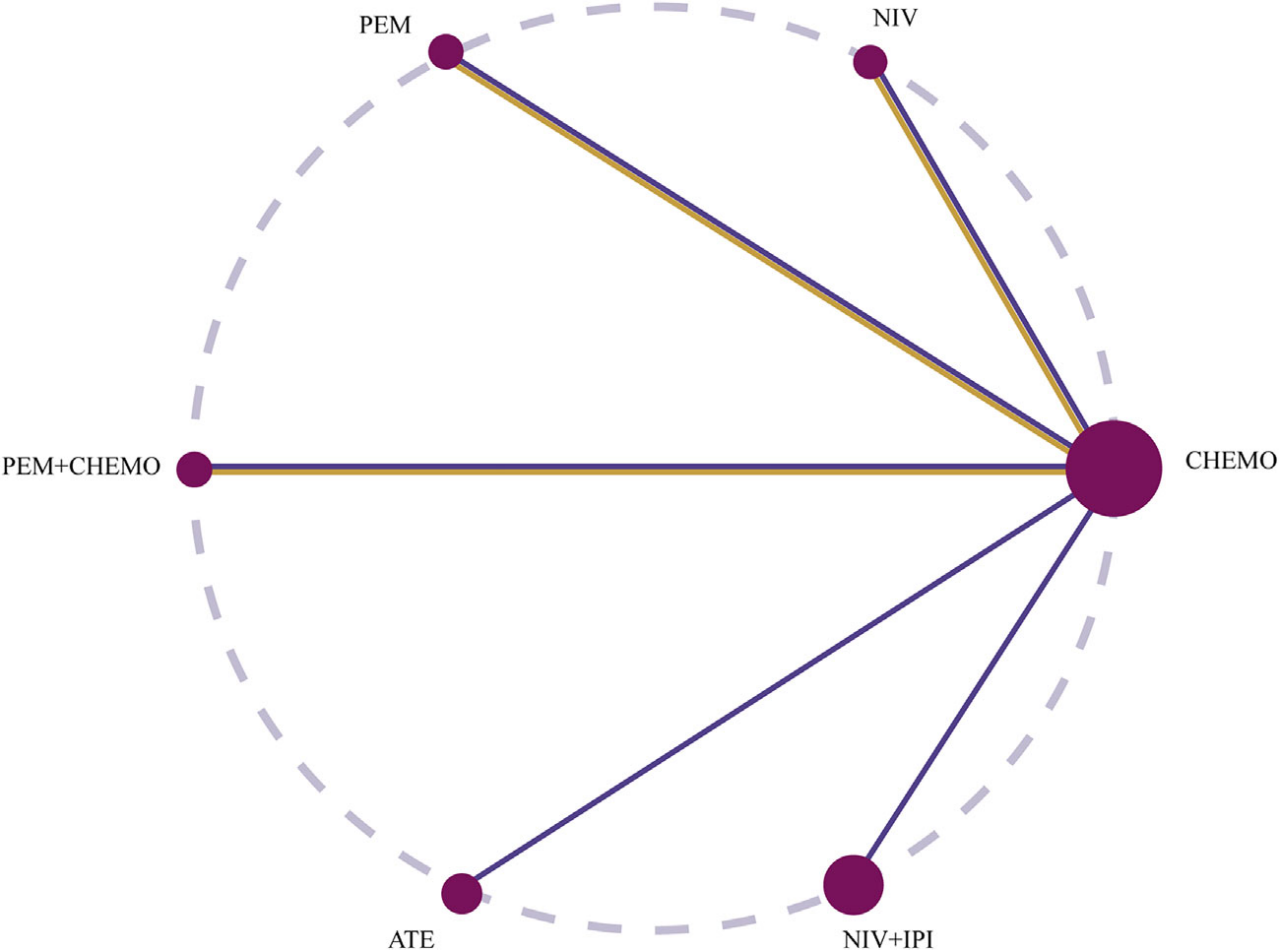
Comparative efficacy of different immune checkpoint inhibitors in patients with advanced or metastatic NSCLC with *KRAS* mutations network diagrams. Purple line: OS network plot, yellow line: PFS network plot. Each circle represents an intervention node in the network and the line width is proportional to the number of RCT trials. Chemo, chemotherapy; Niv, nivolumab; Pem, pembrolizumab; Ate, atezolizumab; Ipi, ipilimumab.

**FIGURE 3:**
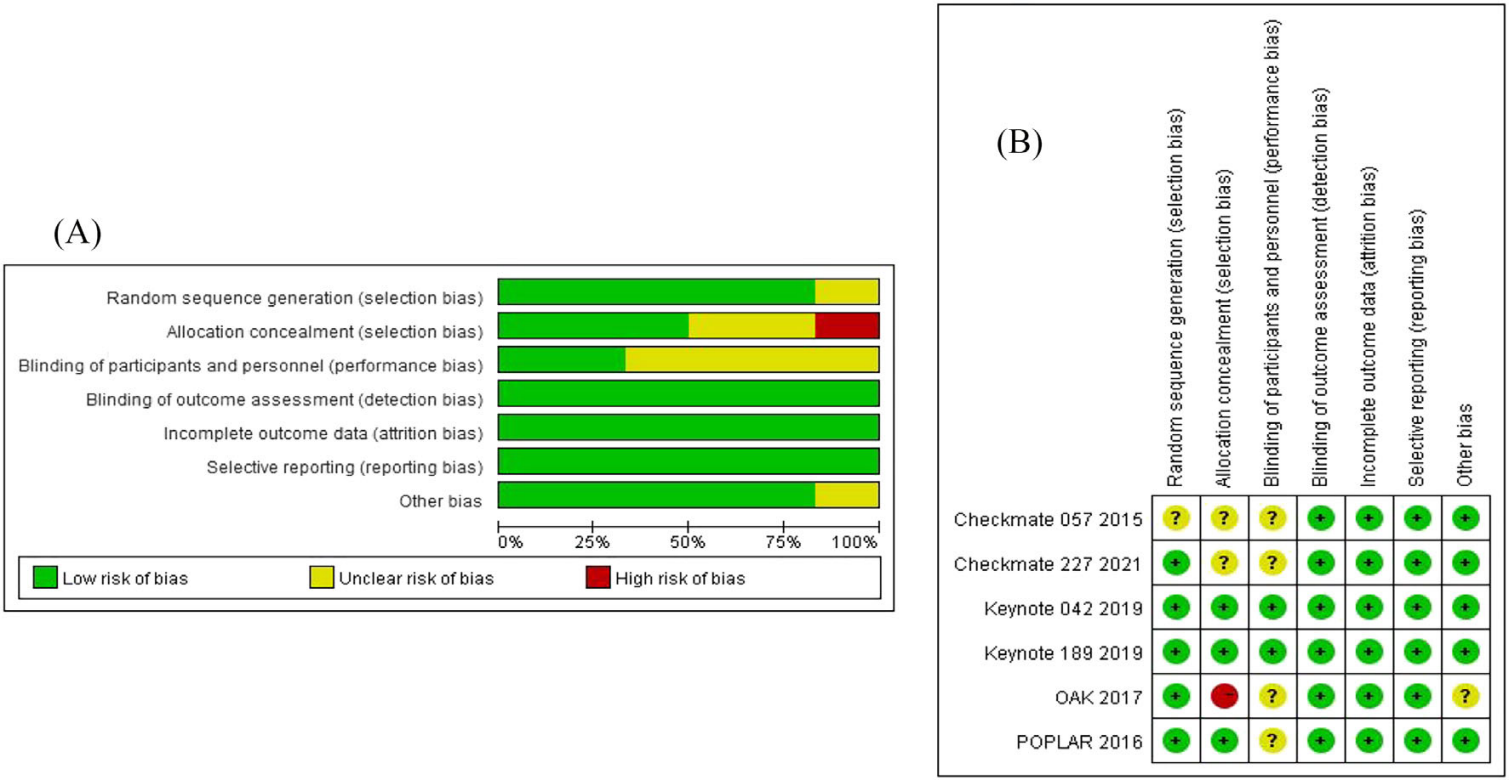
Risk of bias figure.

A total of 5650 articles related to the literature search were identified for this study. After careful examination and screening, six studies were ultimately included.^18^, ^19^, ^20^, ^21^, ^22^, ^23^ The analysis and results in this subgroup might not include the influence of PD‐L1 expression on treatment outcomes. The search and exclusion process can be referred to in Figure 1 for a visual representation of the study selection process. The final inclusion of 469 patients involved six treatment modalities: chemotherapy, ipilimumab plus nivolumab, nivolumab, chemotherapy plus pembrolizumab, atezolizumab, and pembrolizumab. Detailed information is provided in Tables 1 and 2, and the network plot is shown in Figure 2. Keynote‐189, keynote‐042, and checkmate‐227 reported survival data for patients with *KRAS* mutations at the conference.^24^, ^25^, ^26^ Survival data for patients with *KRAS* mutations in the POPLAR trial were derived from a meta‐analysis.^27^ The risk of risk plot is shown in Figure 3.

**TABLE 1:** Baseline characteristics of studies included in the network meta‐analysis.

Study	Auther, year	Registered ID	Sample size		Intervation arm	Control arm
			Intervation arm	Control arm		
Checkmate 057	Borghaei,2015	NCT01673867	28	34	nivolumab(3 mg/kg)every 2 weeks	Docetaxel(75 mg/m2)every 3 weeks
Keynote 042	Mok,2019	NCT02220894	30	39	pembrolizumab 200 mg alone	carboplatin to achieve an area under the curve of 5–6 mg/ml per min plus paclitaxel 200 mg/m^2^ or pemetrexed 500 mg/m^2^.
Keynote 189	Rodríguez,2019	NCT02578680	59	30	pembrolizumab 200 mg, every 3 weeks, for up to 35 cycles; pemetrexed 500 mg/m2 and cisplatin 75 mg/m2 or carboplatin area under the curve 5 mg/min/ml for the first four cycles	saline placebo, every 3 weeks, for up to 35 cycles, pemetrexed 500 mg/m2 and cisplatin 75 mg/m2 or carboplatin area under the curve 5 mg/min/ml for the first four cycles
OAK	Rittmeye, 2017	NCT02008227	26	33	Atezolizumab 1200 mg fixed dose every 3 weeks	Docetaxel 75 mg/m2 every 3 weeks
POPLAR	Fehrenbacher, 2016	NCT01903993	14	13	atezolizumab (1200 mg fixed dose) every 3 weeks on day 1 of each 3‐week cycle	docetaxel (75 mg/m^2^) every 3 weeks on day 1 of each 3‐week cycle
Checkmate 227	Ramalingam, 2021	NCT02477826	88	75	Nivolumab (3 mg/kg) every 2 weeks plus ipilimumab (1 mg/kg) every 6 weeks	platinum‐doublet chemotherapy alone (every 3 weeks for up to four cycles)

**TABLE 2:** Data of studies included in the network meta‐analysis.

Study	*KRAS* mutation status	Hr for OS (95% CIs)	Hr for PFS (95% CIs)	Treatment line
Checkmate 057	Positive	0.52(0.29,0.95)	0.82(0.47,1.43)	2 L
Keynote 042	Positive	0.42(0.22,0.81)	0.51(0.29,0.87)	1 L
Keynote 189	Positive	0.79(0.45,1.38)	0.47(0.29,0.77)	1 l
OAK	Positive	0.71(0.38,1.35)		2 l
POPLAR	Positive	0.94(0.36,2.45)		2 l
Checkmate 227	Positive	0.79(0.55,1.12)		1 l

### 3.2 Pairwise meta‐analysis

**FIGURE 4:**
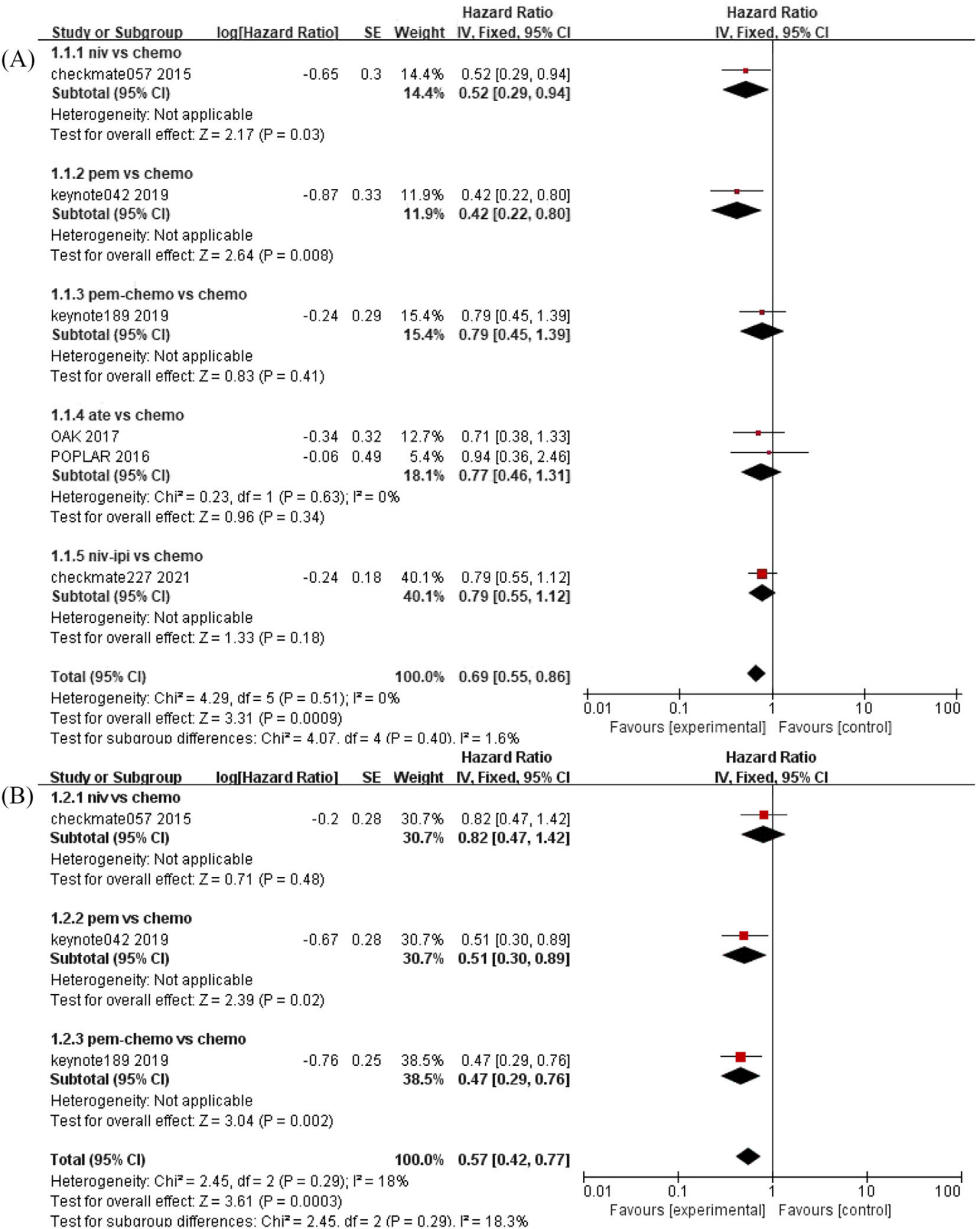
A) Forest plot of OS； B) forest plot of PFS; efficacy of ICIs versus chemotherapy in patients with *KRAS* mutations in advanced NSCLC.

When comparing ICIs with chemotherapy, we conducted a paired analysis (Figure 4).

Six trials report HRs for OS. There was low heterogeneity among the studies (P = 0.51, I^2^ = 0%) and a fixed‐effects model was used. Three trials report PFS with low heterogeneity across studies (p = 0.29, I^2^ = 18%), which were also analyzed using fixed‐effects models. The inclusion of ICIs demonstrated significant improvements in both OS and PFS compared to chemotherapy, with respective hazard ratios of 0.69 (95% CI 0.55–0.86) and 0.57 (95% CI 0.42–0.77).

### 3.3 Network meta‐analysis

**FIGURE 5:**
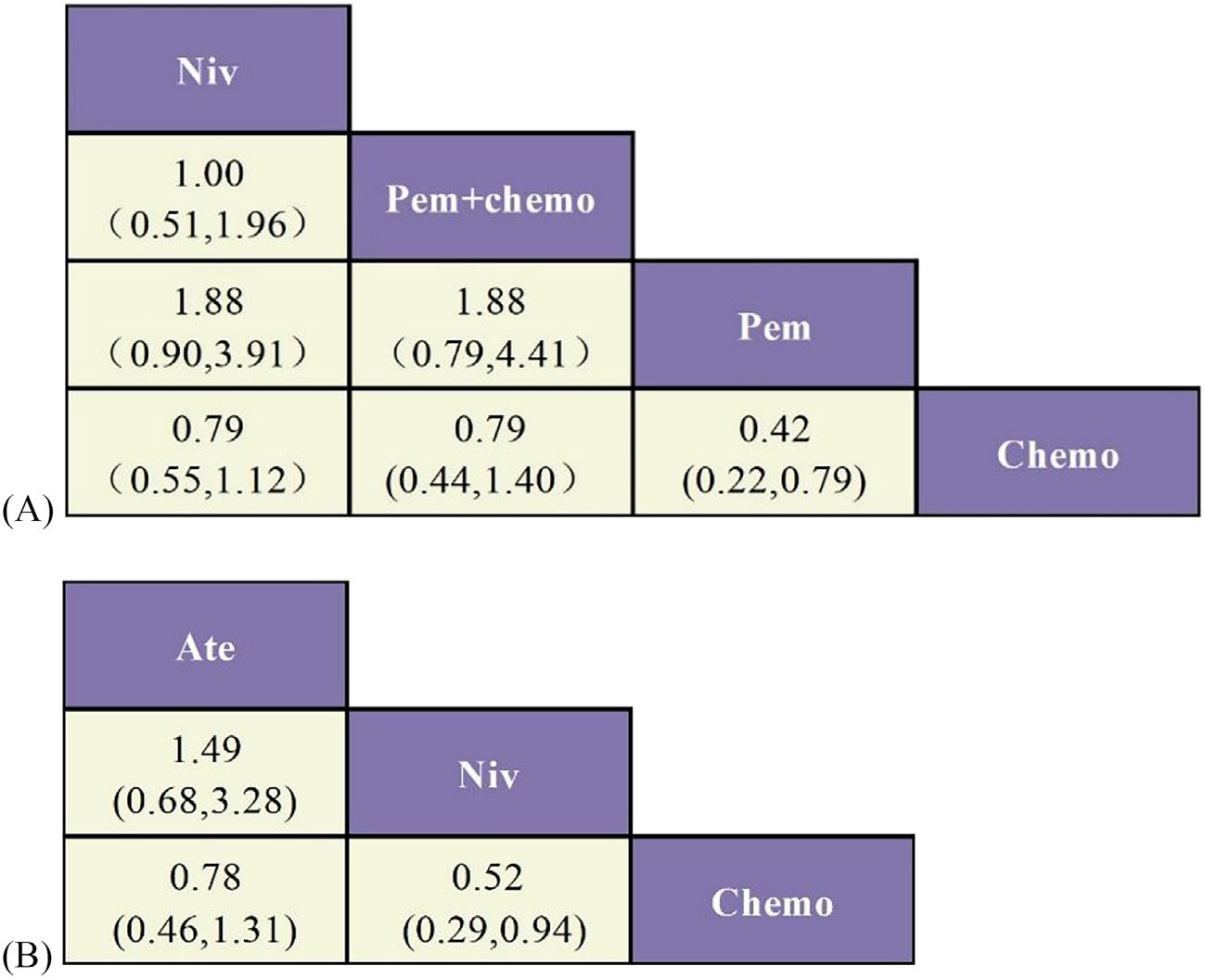
A) NMA of OS for first‐line treatment; B) NMA of OS for second‐line treatment.

**FIGURE 6:**
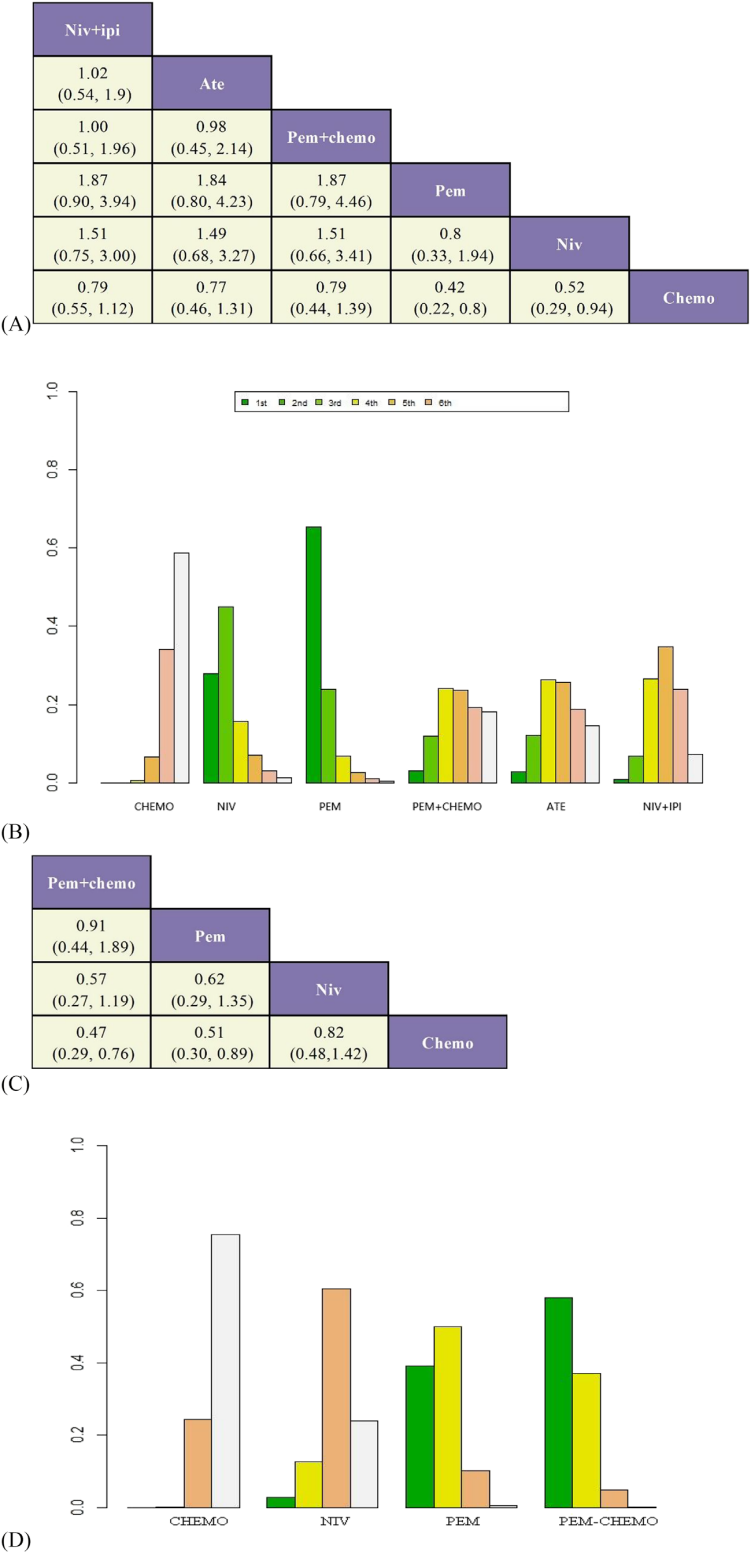
A) Network meta‐analysis of OS; B) ranking of OS; C) network meta‐analysis of PFS; D) ranking of PFS.

A ranking table was created in the NMA (Figure 6). Both ICIs demonstrated superior efficacy compared to chemotherapy. PEM as a monotherapy significantly improves OS and PFS in patients (HR 0.42, 95% CI 0.22 ~ 0.80; HR 0.51, 95% CI 0.30 ~ 0.89). Additionally, the OS of various treatment regimens was compared separately in first‐line and second‐line treatment settings (figure 5). In first‐line treatment, PEM exhibited a significant improvement in patients' OS (HR 0.42, 95% CI 0.22 ~ 0.79), while in second‐line treatment, NIV significantly improved patients' OS (HR 0.52, 95% CI 0.29 ~ 0.94).

### 3.4 Ranking

The effectiveness of treatment options was evaluated and ranked in terms of their probability in Figure 6. Based on our findings, PEM emerged as the most effective treatment option for enhancing OS in patients (SUCRA 65.03%). Additionally, when combined with chemotherapy, pembrolizumab demonstrated superior efficacy in improving PFS (SUCRA 75.39%).

## 4 DISCUSSION

This NMA investigates the optimal treatment strategies for ICIs in NSCLC patients with *KRAS* mutations. OS serves as the primary and most reliable indicator of antineoplastic drug efficacy, forming the foundation for our research and subsequent discussions. The findings from the current study confirm that patients with *KRAS* gene mutations derive greater survival benefits from immunotherapy, which is consistent with earlier research.^27^, ^28^ Patients with *KRAS* mutations exhibit increased levels of PD‐1/PD‐L1 expression. These mutations are proposed to independently affect PD‐1 positivity, leading to an increase in PD‐L1 expression. Furthermore, they can impact specific tumor immune microenvironments (TIME), thus affecting the response to immunotherapy.^29^, ^30^, ^31^ These mechanisms may collectively contribute to the improved survival benefits observed in patients with *KRAS* mutations, enhancing the efficacy of ICIs. Additionally, this study expands on the aforementioned research by ranking the effectiveness of various immunotherapy treatment options. The findings of the study indicate that pembrolizumab emerges as the most effective treatment option for enhancing OS. Furthermore, the combination of pembrolizumab with chemotherapy demonstrates superior efficacy in improving PFS. Among those patients, PD‐1 inhibitors were slightly more effective than PD‐L1 inhibitors. Moreover, the combination of PD‐1 inhibitors with CTLA‐4 inhibitors did not yield any notable improvement in efficacy. This finding is consistent with a previous meta‐analysis conducted in the context of NSCLC.^32^ Theoretical suggestions suggest that PD‐L1 inhibitors may have the potential to achieve more substantial clinical effects compared to PD‐1 inhibitors by blocking both pathways, and it is speculated that the difference between this result and the results of our pooled analysis is due to the ignoring of the role of other cytokines, TIME, and other related factors.^33^

Genetic testing has become increasingly common in clinical treatment due to the development of medical treatment and the focus on personalization and precision. Genetic testing results are frequently employed by healthcare professionals to guide treatment decisions for patients. One of the genes commonly tested is the *KRAS* gene. This study aims to serve as a reference for patients who have undergone genetic testing and know whether the gene is mutated or not. It will help them choose a more accurate and suitable treatment plan, preventing patients from missing the best time for treatment. The researchers also examined the effect of the gene on the drug's efficacy. *KRAS* is a GTPase that plays a crucial role in cell signaling. *KRAS* mutations have distinctive effects on downstream signaling pathways, with different mutations stimulating various signaling cascades. Consequently, the efficacy of the same drug can vary across different mutations in the *KRAS* locus, resulting in differing survival rates. For instance, the *G12D* mutation has been associated with worse overall survival. Understanding these mutation‐specific responses to therapies is crucial for optimizing treatment strategies for patients with *KRAS* mutations.^4^, ^5^, ^6^ The *G12D* mutation in the *KRAS* gene has been found to be negatively correlated with PD‐L1 expression during the establishment of a TIME that is resistant to ICIs. This correlation may contribute to the poor efficacy observed in patients with the *G12D* mutation. Notably, paclitaxel has been shown to improve the tumor immune microenvironment in cases involving the *G12D* mutation. Therefore, the combination of ICIs and chemotherapy, specifically paclitaxel, may lead to improved clinical efficacy for patients with NSCLC who have the *KRAS* ^*G12D*^ mutation.^34^ This emphasizes the significance of tailoring treatment regimens based on individual genetic factors. Furthermore, as targeted therapies for *KRAS* mutations continue to advance, the potential for combining *G12C* gene mutation inhibitors with ICIs as a treatment option for patients with *KRAS* mutations becomes apparent.^7^, ^8^, ^9^

There are several limitations to this study. Firstly, the small sample size obtained from only six randomized clinical controlled trials hinders the establishment of strong and conclusive evidence. Further validation through a larger number of clinical trials or real‐world studies is necessary. This limitation represents the study's primary constraint. Secondly, this study did not explore adverse reactions in NSCLC patients with *KRAS* mutations. The study solely focused on the efficacy of the five treatments and did not analyze their safety profile. Additionally, the study primarily relied on published data regarding *KRAS* mutations and did not specifically examine the survival outcomes in patients with varying PD‐L1 expression. Therefore, there was insufficient discussion on the effectiveness of treatment in patients with *KRAS* gene mutations in the presence of varying PD‐L1 expression.

## 5 CONCLUSION

In conclusion, our NMA compared the efficacy of various treatments involving ICIs for advanced NSCLC patients with *KRAS* mutations. The results showed that PD‐1 inhibitors, particularly pembrolizumab, were more effective in improving patients' OS and PFS, followed by nivolumab based on the rankings. However, in order to provide robust support and validation for our conclusions, additional clinical data is required. Furthermore, continued and sustained follow‐up is necessary to ensure the reliability and long‐term assessment of our findings.

## AUTHOR CONTRIBUTIONS

All authors had full access to the data in the study and took responsibility for the integrity of the data and the accuracy of the data analysis. *Designed research/study*, Lin Zhang and Wei Chen; *performed research/study*, Lin Zhang and Wei Chen; *contributed important reagents*, Lin Zhang and Wei Chen; *analyzed data*, Lin Zhang and Wei Chen; *wrote the paper*, Lin Zhang and Wei Chen; *Writing‐ Review & Editing*, Lin Zhang, Wei Chen, Hongtao Wei, Junxian Yu; *Visualization*, Lin Zhang; *Supervision*, Junxian Yu.

## CONFLICT OF INTEREST STATEMENT

The authors declare no competing interests.

## ETHICS STATEMENT

Not Applicable.

## Data Availability

All data generated or analyzed during this study are included in this published article.
